# A System Model and Requirements for Transformation to Human-Centric Digital Health

**DOI:** 10.2196/68661

**Published:** 2025-04-28

**Authors:** Pekka Ruotsalainen, Bernd Blobel

**Affiliations:** 1 Faculty of Information Technology and Communication Sciences (ICT) Tampere University Tampere Finland; 2 Medical Faculty University of Regensburg Regensburg Germany; 3 First Faculty of Medicine, Charles University Prague Czech Republic; 4 Deggendorf Institute of Technology eHealth Competence Center Bavaria Deggendorf Germany; 5 Department of Informatics, Bioengineering, Robotics and Systems Engineering (DIBRIS) University of Genoa Genoa Italy

**Keywords:** digital health, human rights, privacy, dignity, autonomy, digital economy, neoliberalism, modeling, system analysis, artificial intelligence

## Abstract

Digital transformation is widely understood as a process where technology is used to modify an organization’s products and services and to create new ones. It is rapidly advancing in all sectors of society. Researchers have shown that it is a multidimensional process determined by human decisions based on ideologies, ideas, beliefs, goals, and the ways in which technology is used. In health care and health, the end result of digital transformation is digital health. In this study, a detailed literature review covering 560 research articles published in major journals was performed, followed by an analysis of ideas, beliefs, and goals guiding digital transformation and their possible consequences for privacy, human rights, dignity, and autonomy in health care and health. Results of literature analyses demonstrated that from the point of view of privacy, dignity, and human rights, the current laws, regulations, and system architectures have major weaknesses. One possible model of digital health is based on the dominant ideas and goals of the business world related to the digital economy and neoliberalism, including privatization of health care services, monetization and commodification of health data, and personal responsibility for health. These ideas represent meaningful risks to human rights, privacy, dignity, and autonomy. In this paper, we present an alternative solution for digital health called human-centric digital health (HCDH). Using system thinking and system modeling methods, we developed a system model for HCDH. It uses 5 views (ideas, health data, principles, regulation, and organizational and technical innovations) to align with human rights and values and support dignity, privacy, and autonomy. To make HCDH future proof, extensions to human rights, the adoption of the principle of restricted informational ownership of health data, and the development of new duties, responsibilities, and laws are needed. Finally, we developed a system-oriented, architecture-centric, ontology-based, and policy-driven approach to represent and manage HCDH ecosystems.

## Introduction

Digital transformation is widely understood as a process where digital technology, such as smart sensors, digital monitoring and surveillance tools, ecosystems, the cloud, the Internet of Things, simulation, artificial intelligence (AI), digitalization, and datafication are used by an organization to modify its processes, products, and services to create new ones [1]. According to researchers, digital transformation changes the ways people communicate, learn, and participate. It also raises business and administration efficiency and transforms the organization’s structure and customer relationships [2-5]. Furthermore, digital transformation benefits the whole society by providing better and accessible public services; therefore, it has the power to improve health care and personal health management [3,4,6].

Digital technology is undoubtedly an enabler for digital transformation, but results depend on how and by whom technology is used, as well as the ideas, beliefs, and goals behind decisions. It is often presented as an unavoidable and one-way result of the evolution of digital technology, but this is a misunderstanding. In the business world, digital transformation is increasingly realized using ideas of free market economy, digital information capitalism, and neoliberalism [7].

The use of information technology in health care has a long history. Presently, technologies such as medical imaging, smart medical devices, internet, digital platforms, AI, robotics, big data analytics, blockchain, and smart wearables are widely used [8,9]. Digital transformation in health care is not limited to the use of digital artifacts; it also includes the advancement from data focus to knowledge focus [10]. It is expected to change health care processes and structures; enable personalized services and innovative solutions, such as online remote monitoring, virtual care, digital physician, and health chat boxes; advance diagnosis; and improve planning and management [11]. It changes the patient-physician relationship and responsibilities, including where the health data are processed and by whom, medical education, and patient engagement [3]. It also enables companies to develop new medical devices and innovative digital services for health care as well as commercial devices and applications for personal health and wellness monitoring and management, such as smartwatches, heart rate monitors, blood pressure monitors, electrocardiogram monitors, sleep trackers, smart wearables, and medication reminders [12].

Digital health (DH) is the end result of ongoing digital transformation in health care and health [10]. According to the World Health Organization, it “can revolutionize how people worldwide achieve higher standards of health and access services to promote and protect their health and well-being and have proven potential to enhance health outcomes” [8]. It has the potential to improve patients’ overall health care experience and to offer personalized care everywhere [13].

The ongoing discussion of digital transformation in health care and health is associated with positive promises, such as higher efficiency, lower costs, easy availability and equal access to services, easy personal health management, new innovative services, and better data-driven decision-making [14,15]. These promises are merchandized to decision makers and society as an inevitable outcome of technological determinism aimed at digitalizing human life, body, and mind [16]. At the same time, discussion of possible negative consequences is largely neglected, and they are presented as things to be solved in the future. According to researchers, digital transformation, datafication, digital tools, and algorithms have already enabled almost unlimited data surveillance and tailored nudging of people. It has provided a huge amount of power to data collectors and algorithm owners, and its negative impacts on health care and health are immense. This has raised concerns about human rights, privacy, and autonomy [17]. For managing this problem, the IEEE P7008 Standard for Ethically Driven Nudging for Robotic, Intelligent, and Autonomous Systems should be deployed.

In this paper, we aimed to present detailed insights into the digital transformation of health care and health to DH and develop a new model called human-centric DH (HCDH) that provides most of the promised benefits of DH without the loss of human rights, privacy, dignity, and autonomy.

## Holistic View of DH

**Figure 1:**
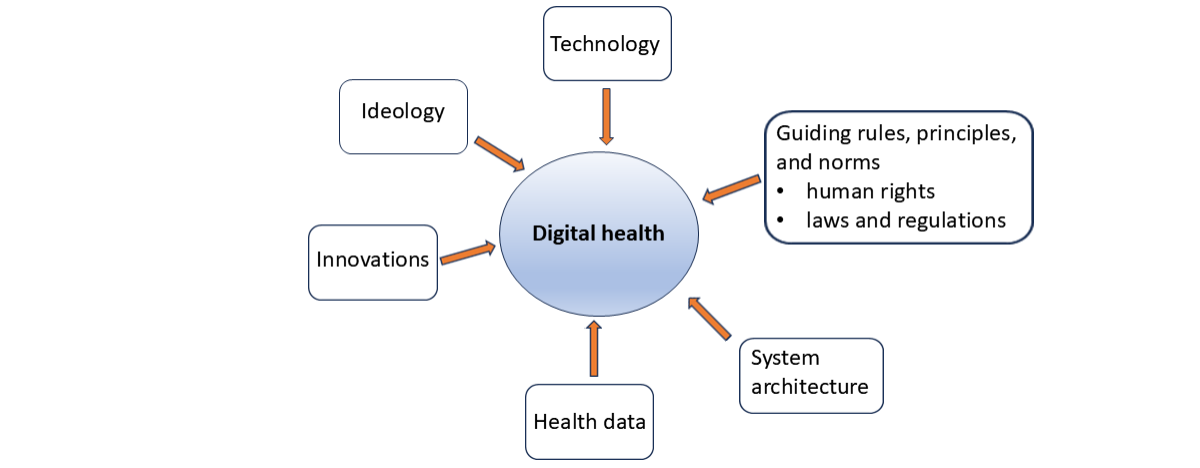
A framework for transformation of health and health care to digital health.

In this paper, DH was studied as a holistic system. Up to 560 research articles published in major journals covering a wide range of topics such as human rights, dignity, privacy and autonomy, neoliberalism, digital transformation, digital technology, DH care, DH, and digital twin were reviewed in detail. We analyzed ideas, beliefs, and goals guiding the transformation of health and health care to DH and their possible consequences on information privacy, human rights, dignity, and autonomy. On the basis of the analysis performed, a framework of 6 views that impact the nature and functions of DH was developed (Figure 1). Ideologies, that is, beliefs and opinions, form the base of transformation. The “data view” is derived from the fact that successful DH requires a great deal of health-related data to enable its services. Guiding rules, principles, and norms (eg, human rights, laws, and regulations) both direct and set restrictions concerning what data can be collected and used. The “architectural view” indicates that DH is an information system that requires a conceptual, organizational, and technical architecture. Finally, technological, architectural, and organizational innovations are enablers for the realization of DH as the system that fulfills its ideas and goals.

## DH Concept

DH itself is a concept with many definitions. In a review by Fatehi et al [18], they found 95 different definitions of DH. A narrow health care–focused definition expresses that DH is “the cultural transformation of disruptive technologies that provide digital and objective data accessible to both caregivers and patients” [19]. According to Yeung et al [20], DH is a broad term covering the application of digital technologies in the context of health based on the definition for health (“a state of complete physical, mental, and social well-being and not merely the absence of disease or infirmity”) by the World Health Organization [21]. This definition expands the concept of DH to cover health determinants other than health care services (eg, environment, genetics, and social relations) [22]. It also encompasses uses of digital technologies, such as the internet, big data analytics, AI, and robotics [20]. Because access and use of health care services have a much lesser impact on human health than other health determinants, personal wellness lifestyle management, and digital technology that is used to collect and use personal health information should also be included in the concept of DH [20,23]. In this paper, the definition by Fatehi et al [18], “Digital Health is proper use of digital technology for improving the health and well-being of people at individual and population levels, as well as enhancing,” is used [18].

## Economic Ideologies and Digital Transformation in Health Care and Health

In spite of health care’s strong professional ideology, its service providers are increasingly looking not only for better service accessibility and higher quality but also higher efficiency, optimal allocation of resources, lower costs, and possibilities of digital technology. This indicates that economic ideologies are increasingly transforming health care.

Classical capitalism is an economic model based on private ownership of property and business. Its goal is the maximization of its own (monetary) utility without responsibility for possible negative consequences [24]. Free market and weak state control are its additional elements. According to Sadiku et al [25], digital capitalism (ie, platform capitalism, informational capitalism, and surveillance capitalism) is a natural extension of classical capitalism [15]. It uses digital technologies, such as the internet, AI, digital algorithms, blockchain, digital platforms, and the Internet of Things for data collection, processing, and sharing [26]. Therefore, data about people’s activities, behaviors, lifestyle habits, and emotions are both a meaningful commodity and product, and it is expected that in the long run, all aspects of human life can be quantified and transformed into information for private or public benefit [26,27].

According to McGregor [7], neoliberalism is the logical evolution of digital capitalism. Neoliberalists believe that social good will be maximized by maximizing “the reach and frequency of market transaction,” and a “good life” arises from “free” individuals under a free market system [28]. At the ideological level, neoliberalism’s aim is the commodification of goods, services, and knowledge, and reducing most aspects of human lives into “goods” (ie, datafication) [28]. Core ideas in neoliberalism are privatization of public services, deregulation, marketization, self-regulation, strong private property rights, free markets and trade, the removal of regulatory barriers to commerce, and maximizing efficiency. Furthermore, the government’s main role is to boost private-sector business and weaken regulations. Personalized data are collected to generate behavioral changes. One of neoliberal goals is to commodify traditional public services [29]. Neoliberalists replace the concepts of the public good and the community with individual responsibility based on self-interest and individualism [7]. It is also assumed that humans will always try to favor themselves, and that they do not feel the need to consider the consequences of their actions toward others [7].

Because a market economy and neoliberalism are prevailing and dominating ideologies in many sectors of society, a likely scenario is that DH will be built around beliefs and goals of a market economy and neoliberalism. In this paper, this scenario (“neoliberal DH”) is used as an example. In health care and health, this means that services are organized using the economic free market model and privatization, monetization of patient data, and datafication of all health data as the most effective solutions to organize and offer equal health care services and treatments for all [7]. Another idea is consumerization, where people are understood as rational health consumers having the ability and capacity to self-manage their health and sickness. Commodification is also necessary for better care and lower cost, especially the commodification of the content of regulated health care records, as well as other personal health-related data. The idea of health care as a public good is replaced by the idea of individual responsibility for health and health care as private goods. Datafication of all health data is necessary for disease prediction, personalized care, early detection of diseases, modification of people’s health behaviors, creating digitally engaged patients, and earning money [30].

## Principles and Guiding Norms

According to Stanley et al [31], normative ethical theories and religious traditions offer general moral principles for people to follow. The United Nations’ human rights are universally accepted and used in many international and national laws as guiding principles and norms for human behavior. Human rights are basic rights and important for human flourishing, individual freedom, and social justice [32]. Human rights and dignity are 2 interlinked concepts. In the preamble of the United Nations Declaration of Human Rights (UNDHR), human dignity is the inherent dignity, that is, human rights are derived from human dignity, as human dignity is the foundational principle of human rights and the wellness of society. They are needed to prevent injustice, exploitation, discrimination, and inequality [33,34]. Examples of human rights are the right to life, equality before the law, liberty, personal security, the right to education, and the right to free movement. Among the 30 articles of the UNDHR, articles 12 and 18 are especially meaningful for DH [35]. According to article 12, “No one shall be subjected to arbitrary interference with his privacy, family, home, or correspondence, nor to attacks upon his honor and reputation. Everyone has the right to the protection of the law against such interference or attacks,” and according to article 18, “Everyone has the right to freedom of thought” [35,36]. Furthermore, governments have obligations to protect human rights everywhere, including in the digital context [2,35,37].

Dignity itself is a multidimensional and vague concept. At the general level, dignity concerns “moral status involving inherent unearthed form of worth that is independent of the interest of others and not based on one’s merits” [38]. The idea of the intrinsic human worth of honor or respect is global and exists in some way in all cultures [34]. Dignity is also linked to personal characteristics and collective behaviors in social relations. Human dignity gives moral weight to the idea that people cannot be owned as property by others [39]. Furthermore, the European Union Charter of Fundamental Rights declares that human dignity is inviolable and must be respected and protected [40].

Autonomy includes concepts such as self-rule, self-determination, individuality, and independence [41]. Furthermore, it addresses the right of people or a group to govern themselves or to organize their own activities [42]. According to the UNDHR, bodily autonomy is a fundamental right. Individual autonomy is widely understood as the capacity to be one’s own person, to live one’s life, and not to be the product of manipulative or distorting external forces [43]. In liberalism, autonomy implies the ability to reflect wholly on oneself, to accept or reject personal values, connections, and features [43].

Privacy is a vague, emotional, and contextual concept with social and technical dimensions [44]. It is dynamic because people’s privacy expectations and needs vary situationally. Privacy is also one of the human rights. The UNDHR expresses that “No one shall be subject to arbitrary interference with his privacy” [35]. Autonomy and privacy are closely interconnected. According to Citron [45], without personal autonomy, there is hardly personal privacy, and privacy ensures dignity. Furthermore, we must understand privacy as a moral right, human right, and legal right, and that invasion of privacy is a violation of human dignity [45]. Because privacy is not an absolute right, it is not uncommon to think that other values, such as the public good or common good, can supersede it.

Traditionally, the focus of privacy has been the individual, the individual’s space, and individual interests. It has been seen as a right to control what others know of us, and the ability to control data collection, use, and disclosure [44]. Control of data flow and use of data are dominating mechanisms to balance others’ “need to know” and individuals’ intention to limit what others know of us [39]. Contextual privacy by Nissenbaum [46] is a new approach that allows information to flow freely inside the context and between contexts following “filtering rules” defined by stakeholders in the context. Privacy also has a social dimension, enabling the management of the social boundaries and freedom of thought, including limiting the power of governments and data companies regarding the use of data [45,47].

According to Wang et al [48], digital privacy is a “selective psychological and technical control of access to the digital self in the form of online profiles, personal data, and digital assets.” Internet privacy addresses the level of privacy a person has on the internet. Online privacy refers to the right to privacy in online situations. New ideas about privacy include group privacy, privacy as ability, privacy as property, and mental privacy. The authors state that privacy remains an abstract concept until people have the ability and power to express personal privacy preferences in real-life situations.

According to Abernethy et al [11], the prevailing pervasive, dynamic, and virtual digital world, where passive and online data collection on people’s activities and behaviors takes place routinely, and where people self-disclose their personal information, has challenged most of the traditional privacy models. The digital world is virtually without privacy boundaries, and information collection, sharing, and self-disclosure make us transparent to others. This and AI transform how privacy is understood and change the level of actual privacy we have [49]. According to researchers, in the digital world, privacy is not only needed to protect personal health-related data but also to preserve one’s human rights, dignity, and autonomy [39,48,50].

## Laws and Regulations

Different countries have national regulatory frameworks for health care regulating the collection, use, sharing, and storing of patient data and patients’ information autonomy. Thus far, DH-specific laws have been missing, and therefore, existing general and health care–specific regulations can be used where appropriate. In a former paper [51], the authors stated that the current regulatory environment is insufficient and outdated for digital environments. Brantly and Brantly [52] argued that the regulations fail to place the patient or person and his or her well-being at the center, and the expected benefits of needs of digital markets increasingly override the data subject’s privacy, dignity, and autonomy [52].

## Health Data and Ownership

The power and benefits of DH are strongly dependent on the availability and quality of health data coming from different sources, such as health care activities, smartphones, health apps, and social media, frequently generated by patients themselves [53]. There are different opinions regarding which data should be classified as health data and the nature of health data. Currently, it is unclear whether health data should cover lifestyle and well-being data and sensor data regarding an individual’s daily behaviors, habits, and preferences [54]. A narrow view on health data includes only data generated by health care professionals and medical devices, that is, the content of the regulated patient record. A wider view recommends that all kinds of health-related data, which come from inside and outside the regulated health care domain and can be collected, processed, stored, and interlinked, should be included in the discussed health data space [55,56]. In this wider view, sources of health data include regulated health care service providers, nonregulated wellness firms, governments, internet firms, social networks, industry and researchers, and people and patients who voluntarily generate and disclose health-related data.

A similar dichotomy also exists in regulations. The European Union General Data Protection Regulation (EU-GDPR) treats health data as a special category of sensitive information and uses a broad view of health data by defining it as “personal data concerning health” that includes “all data pertaining to the health status of a data subject” [57]. Recital 35 of the EU-GDPR details this definition by defining health data as “any information on a disease, disability, disease risk, medical history, clinical treatment, or the physiological or biomedical state of the data subject” [58]. In Contrast, in the United States, the term “protected health information” is used for health data, covering only entities directly related to health care operations [55,59].

Opinions concerning the nature of health data also vary. Health data are understood as public goods, common goods, commodities, or personal properties [60]. Some argue that health data should be common goods that enable economic growth and new research innovations [61], and others propose the open data model as the future way to go. Martin and Begany [62] argued that government agencies should implement an open health data model for better efficiency and for benefits, such as improved health literacy, data-driven changes in health care delivery, consumer engagement, and community development. In contrast, Verhulst [27] stated that health data cannot be treated solely as a public or private good.

The ownership of health data is also a critical question. Opinions are shared regarding the concepts of ownership and property of health data. In principle, the owner of health data can be the data subject or patient, a public or private organization, or the ownership can be shared. Nowadays, companies (eg, internet giants and mediators) that have used remarkable resources to collect, organize, and use health data often expect that “they either fully own or have full and complete rights to the data” [30,47]. This view is also supported by the EU in its regulation that grants property rights to those “who collate large compilations of digital data and other data” [63]. As part of the digital single market strategy, the European Commission sees that ownership, such as property rights and database rights, poses a threat to the development of an EU data market, and big data should be as freely accessible as possible [64].

A benefit of personal ownership of health data is that it gives the person or patient the power to maintain privacy and autonomy in different contexts and situations, and to restrict unnecessary data collection. Shared ownership of health data means that, in a specific context, a group of users have the right to use it freely. According to Lipton [30], the models of legal ownership require human effort to create those data, and because personal information is not created by a person, it cannot be owned. Some researchers have proposed that in the digital world, health data should be a special and legal intellectual property of the data subject [65].

## Organization, Architecture, and Technology

A DH system can be built using different organizational solutions and models, such as public-private partnerships, data trust models, data cooperatives, data collaboratives, and health data market models [66]. DH can be a public-private partnership system or a multistakeholder health ecosystem, where the main players are commercial organizations owning not only health data but also algorithms, analytic tools, and personal health applications supporting self-diagnosing and self-care, and where the role of public health care is small. Data collaboratives are initiatives for data collection, sharing, and processing. The health data cooperative (eg, a health data bank) enables the users to collect, store, manage, use, and share health information to analyze it, and to conduct big data analytics [67]. In the data cooperative, health data are equal properties of all its members, and it offers worldwide accessibility to data [67,68]. The platform for health data marketplaces can be owned by the government or a private organization [69]. Its business model is built around personalization and the commodification of incoming data. Data trusts are intermediaries where the owners or managers have the responsibility to act in the interest of beneficiaries [39]. An interesting new solution is based on the fiduciary duty model [70,71]. According to Hashiguchi et al [70], the legal fiducial duty model can be used in public-private partnership solutions.

From the technological point of view, DH is a complex sociotechnical information system. Different architectural approaches and different hardware and software solutions can be used to realize DH systems, such as a digital ecosystem and platform, blockchain, edge computing, confidential computing, federated learning system, digital algorithms, the Internet of Things devices, smart sensors and wearables, and health applications, among others [26,60].

## Technological Innovations

New technological innovations are coming soon into practice, such as digital phenotyping, biomedicalization, use of digital biomarkers, synthetic biology, gene manufacturing and editing, DNA tailoring and printing, brain computer interface (BCI), use of digital biomarkers, generative AI, AI-enabled robotics, quantum computing, and digital twin technology [72]. They offer new possibilities for DH and in the development of new services and tools, such as online patient monitoring, self-reporting, AI-assisted care robots, AI-based DH assistants, and conversational agents using generative AI [73].

Digital biomarkers are quantifiable physiological and behavioral data collected by personal digital devices, and digital phenotyping involves the collection and quantification of individual-level characteristics. Both are used to explain, influence, and predict health-related outcomes [74]. The focus in biomedicalization is behavioral and lifestyle modifications of humans [75,76].

BCI uses implanted or outside-the-body monitoring devices to measure, quantify, and interpret human behaviors and even thinking [77]. In BCI, direct communication link between the brain and a computer is used to enable access to our mind and even to upload our brain with new data [78]. Currently, BCI allows recording of brain activity and translation of brain signals to text [79].

Th digital self is a virtual model of a person’s physical characteristics and provides a larger view of an individual as a computer representation in the digital domain based on their personal data [80]. It can be used to simulate a person’s behaviors [81]. A digital patient twin (DPT) is a simulation model of a patient constructed from patient data, genetic data, personally generated health data, data from monitoring devices, and population data [81]. Generative AI, digital phenotyping, and digitalization of the human body and behaviors enable the creation of the DPT. It offers huge opportunities for “precision medicine” by enabling the simulation of how the body responds to treatment and medication.

The digitally engaged patient (ie, the empowered or activated patient) approach assumes that more information leads to improved quality of care and economic efficiency. Digitally engaged patients use personal monitoring tools to self-monitor personal biomarkers, self-care of their own illnesses, and manage their personal health [4]. It is expected that in the long run, digitally engaged patients can develop health care provider–level expertise concerning their diseases.

Because new technologies offer unprecedented, detailed insight into the human body and mind, they also raise new concerns for human rights, privacy, dignity, and autonomy [23,82].

## DH: Concerns to Human Rights, Dignity, Privacy, and Autonomy

### Human Rights and Dignity

Digitalization of all aspects of the human body, behaviors, emotions, feelings, thinking, and mind enables the creation of personalized repositories of the self, owned either by the government or private organizations. That kind of ownership (also known as owning a digital version of a person) can be understood as digital slavery. According to human rights, “slavery shall be prohibited in all their forms” [35]. Therefore, digital slavery is against human rights. Because DH twins are forms of a digital self, creating and trading them without permission is against human rights.

The ability to own health data and use AI technology, digital nudges, and conversational agents gives public and private organizations the ability to control and manipulate an individual’s health behaviors, emotions, mind, and thinking online and offline in a way that can cause the loss of dignity [72,83]. This can cause harm and interfere with personal dignity and autonomy. It is generally wrong and contradicts human rights.

In the digital world, many social processes can be used to violate dignity. In an article, Jacobson [84] named 24 such kinds of processes. Jacobson argues that the following among them are relevant for DH: dismissing intrusions to personal boundaries; threatening an actor as a thing, not a person; limiting an actor’s ability to direct his or her own life; manipulating for material gain or psychological advantage; seeing an actor not as a unique individual, but only as a member of a collective; pressure or bypassing reasons; and treating a person as thing [84].

Nowadays, an important problem is that private companies only have a moral duty to respect and protect human rights [85]. Furthermore, weakening the state’s role in DH weakens its ability to protect human rights and dignity.

### Privacy and Autonomy

Digital transformation and DH have already started to change the way privacy is understood. According to Suleyman and Bhaskar [72], autonomy and privacy are challenged by pervasive online data collection and surveillance systems and the use of AI. Today, it is widely accepted that publicly available personal information and metadata require no protection, and firms do not have the responsibility to control the secondary use of health data that erodes privacy. Companies also often see that a high level of privacy reduces their economic gain. Therefore, companies’ primary interest is the ability to freely collect and use health data and not to protect privacy and autonomy [45].

Currently, a person has little or no power to control the collection, use, and sale of personal health data [86]. Furthermore, the ownership of health data is increasingly transferred to AI system developers [72]. This gives them the power to change the way privacy and autonomy are understood and implemented, and thereby to reduce data subjects’ privacy and autonomy. Another complaint is that many internet service providers do not have a strong privacy mechanism in place, and information platforms are unaccountable for vulnerabilities in their information systems, and privacy and security concerns that they generate [25,87]. Furthermore, self-reporting, online data collection, surveillance, lack of transparency and accountability, and the mining of health data raise autonomy and privacy concerns, and algorithmic manipulation erodes human autonomy and alter how it is understood [53].

### Laws and Regulations

Broadly, current laws and regulations have many weaknesses. They are inadequate and fail to sufficiently protect privacy [45,86]. Nowadays, laws insufficiently regulate how companies can use data [66], and they use a too narrow definition for health data. In current laws, privacy is not correctly managed, and it is balanced against other rights and public goods. Consequently, other rights, such as national security legislations and legitimate interest to know, regularly override privacy. Furthermore, current privacy laws do not regulate the use of inferred and derived data products and do not set concrete responsibilities for organizations collecting and using health data. Laws are also poorly prepared for new privacy and autonomy risks existing in DH and raised from the use of new technology. For example, the EU-GDPR protects only the privacy of a natural person who is alive, and it neglects to protect inferred data, personal profiles, the data donation model, and the use of data after death [15]. Furthermore, it protects biometric data (eg, fingerprints, facial images, and voice) and physical and physiological characteristics of a person, but not induced informational products and digital and simulated models, such as the DPT. In the EU-GDPR, legitimate interest means that a company can process sensitive data as long as a person’s fundamental rights and freedom are not seriously impacted, and the legitimate interest does not require a specific purpose nor the users’ consent to collect and process data [30]. According to Cohen [87], legitimate interest does not fulfill the requirements of transparency, data minimization, purpose limitation, and accountability.

The EU AI Act is a step forward in regulating the use of AI. The act has 4-level classification of AI risks (eg, unacceptable, high, limited, and minimal risk). AI systems that use technology, such as subliminal manipulation, social scoring, or predictive tools, and pose a clear threat to safety and rights belong to the high-risk group and should be banned [83]. Instead, high-risk AI systems should be regulated, and limited-risk systems require just transparency [88]. The weakness is that other kinds of manipulation, such as trickery, pressure, or bypassing reasons and treating a person as a thing, require only transparency [89]. A general problem is that there is currently no consensus on which forms of influence are manipulative. For example, it is unclear what is an acceptable level of manipulation of a person’s health behaviors for better personal health [83]. Furthermore, the term “risk” is error-prone because in real life, it is almost impossible to measure the actual risk for privacy and autonomy [90]. In the United States, the new AI Bill of Rights looks data privacy through design choices, such as protection by default, and supports algorithmic transparency and the users’ ability to opt out of automated systems. Unfortunately, this bill is not binding and does not regulate private companies [83].

According to researchers, the current laws are too weak in protecting privacy and unable to manage problems created by digital technology and digital transformation based on ideas of digital capitalism and neoliberalism [91]. Furthermore, laws are based on an individualistic privacy model that is too narrow assumption [47].

### Organizational Models and the Use of Digital Technology

DH can be realized using different organizational approaches, such as public-private partnerships, data trust models, data cooperatives, data collaboratives, and health data markets [66]. However, none of them guarantee privacy and individual autonomy. Challenges in the health data marketplace model cover trust, privacy, and security. Regarding data trust, the challenge is the control of health data use as well as the possible misuse against human rights and dignity. In DH ecosystems, patient data can be a commodity and different stakeholders, such as universities, public health care providers, big and small technological companies, and nonregulated private organizations, can use and share health data freely. Furthermore, the “ecosystem” where stakeholders have different business needs, regulations, and responsibilities raises concerns of data misuse, loss of privacy and autonomy, and endangers dignity and human rights [12].

The use of digital technologies promises huge benefits to improve health and well-being; however, it also raises threats to privacy, autonomy, and dignity by altering the ways individuals, governments, and firms interact [92]. Health AI needs a lot of training data, including reference data from healthy persons to perform a detailed analysis, create profiles, and develop tailored predictions and simulations. This and the fact that the use of AI applications in care makes patient data part of the training data leads to loss of privacy and autonomy. Currently, AI systems are “black boxes” without transparency and the ability to explain how their decisions are made. Furthermore, they lack the capacity to recognize causality and ability, and to answer questions “why” and “how.” Many of the promises of AI are just beliefs [7,93]. This makes the forecast of negative consequences unpredictable [71]. Nowadays, information systems and AI algorithms can access a large amount of health data without having the necessary safeguards, and they lack accountability [39]. Furthermore, transparency alone is insufficient because it fails to protect people against emotional manipulation [94,95].

New technological innovations create new challenges to human rights, dignity, privacy, autonomy, and equity. For example, the brain computer technology creates danger to freedom of thought and autonomy. Generative AI used in health can produce much misinformation (hallucinations) and raise concerns about the fair use of data and data ownership. In the future, AI technology may be able to infer our thoughts, and neurotechnology could decode our emotions, and perhaps soon our thoughts, and in this way, our mental freedom would be in danger [96]. Digital technologies, such as nudging, manipulation, and conversational agents, enable the manipulation of people’s health behaviors, emotions, and mood, and in this way undermine autonomy and dignity [97]. Furthermore, machine learning and AI can destroy privacy by enabling tailored surveillance and reidentification [55]. Crucial and currently unsolved questions include who can create and use DPT, whether a health care provider can make and modify it without a patient’s informed consent, and for what purposes it can be used without a care relationship. In the future, it is possible that DPT may be increasingly the subject of care without a patient-physician relationship. This can destroy autonomy and lead to new paternalism and unknown or undefined responsibilities [97].

### Concerns in Neoliberal DH

In this paper, neoliberal DH is studied as a possible solution for DH. According to researchers, the belief that it is the most effective solution and offers the best health outcomes and lower costs is not an “objective truth” [47]. The maximizing single service provider’s economic gain will not automatically maximize the quality of service and offer the lowest total costs. Furthermore, individuals who have money, social position, and relations have the best ability to use services and receive benefits. The result is that neoliberal DH can be harmful to the population’s health and well-being and can negatively impact health care affordability and quality [91].

Neoliberal DH has various impacts on human rights, dignity, privacy, and autonomy, as shown in Table 1.

**Table 1:** Impacts of neoliberal ideas on human rights, dignity, privacy, and autonomy in digital health.

Idea or goal	Meaning in digital health	Impact on
Digitalization of all information	A person’s life, emotions, and health behaviors are digitalized.	Privacy, human rights, and dignity
Privatization and monetization of health data	Health data are available for all and sold for money. The person has no possibility of knowing how health data are used and by whom. Health data are shared with private artificial intelligence developers, and they become part of the big data training bases.	Privacy, dignity, and autonomy
Commodification and privatization of public health services	Weakens the role of public services.	Privacy, human rights, and autonomy
Self-regulation, deregulation, and minimization of the state’s role	Protective restrictions for health data misuse and quality norms are weakened. The power of private organizations has increased.	Autonomy and privacy
Transferring responsibility to a person	Makes the person or patient a consumer. A person or patient has the responsibility of care, personal health, and for self-monitoring.	Autonomy and privacy
Private ownership of personal health data and digital products derived from it	Private organizations own health data, personal profiles, and digital health twins derived from them; enables tailored manipulation of a person’s health behaviors.	Human rights, dignity, and privacy; autonomy and privacy
Minimizing private service providers’ responsibility	Private organizations lack the responsibility for the negative consequences of the use of health data.	Human rights and privacy
Digital services substitute human contact	Caring data, profiles, forecasts, and digital patient twin.	Human rights and dignity

According to Wong [39], the commodification of public health services and health data, especially when it is linked to monetization and privatization, creates meaningful human rights, privacy, and autonomy concerns [39]. Commodification and privatization of health data move the ownership of health data to private organizations that give them the power to change norms and use health data for the development of new business, services, and tools [98]. Furthermore, the monetization of health data enables selling it to private and public users operating in different environments and jurisdictions. It increases the number of actors collecting, using, and storing health data in different contexts, and powerful private organizations (eg, internet giants and mediators) acquire the power to reshape laws, regulations, and standards for their own sake [87]. The result is increased risk of data misuse, unlawful destruction and modification, unauthorized disclosure or access, and use of data for other purposes than for which it was collected [85].

Defining people as consumers enables the transfer of responsibility of health and care to the person in the form of self-care, home care, home hospital, self-tracking, digitally engaged patient, and self-control, and at the same time reduces health care cost [47]. The problem in self-responsibility is that many exogenous factors impacting personal health and wellness are largely beyond individual control, such as environmental and air pollution, genetic predispositions, biological factors, neuropsychology, genetics, and epigenetics, as well as factors related to a person’s economic and socioeconomic status [99]. Furthermore, researchers have shown that the idea of a rational human that is personally responsible for own beliefs, intentions, health behaviors, and actions is only a belief, and people hardly have the knowledge and power to make rational health decisions [47]. Therefore, the idea of placing the responsibility for health and wellness on individuals is a mistake [99].

## A Road to HCDH

**Figure 2:**
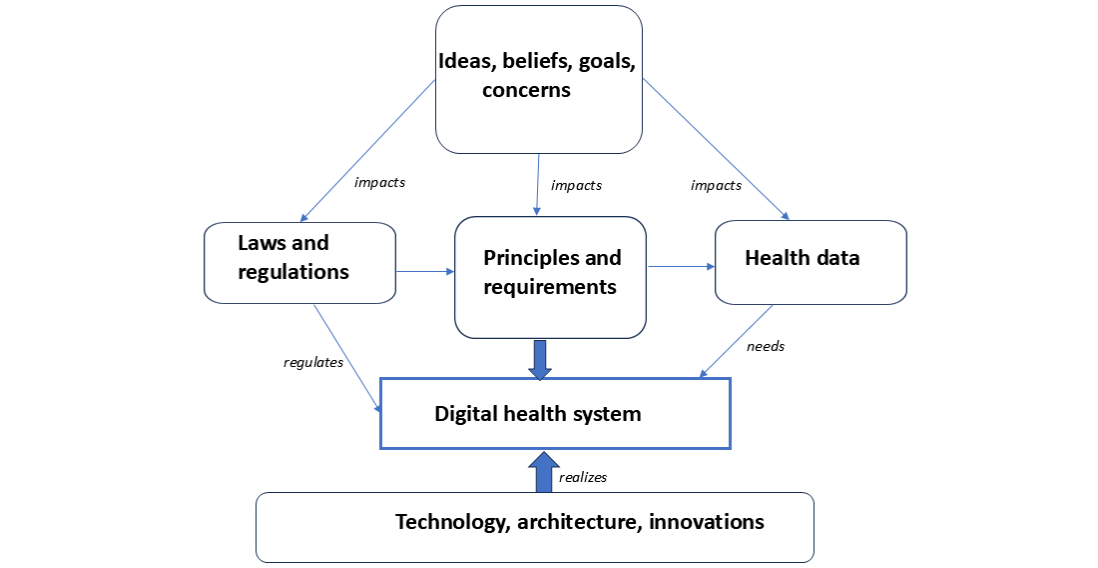
System model for creation of human-centric digital health.

The ultimate goal of the HCDH model is to support good health and wellness for all and not to produce harm and maximize profit. It sees DH as a sociotechnical system whose core elements are ideas, goals, principles, and regulations. The term “human-centered” means that the patient’s or person’s needs for human rights, dignity, autonomy, and privacy are acknowledged, and the person or patient is the stakeholder in the DH system, having the power to use autonomy to set personal privacy obligations. The model highlights that the main challenge in creating HCDH are the formulation of fundamental ideas and goals, understanding health data in the right way, and the creation of principles, new laws, and requirements. System thinking and modeling methods were used to develop the model. The model uses broad definition for DH provided by Fatehi et al [18] and takes a wide approach to health data, as depicted in Figure 2.

In this model, ideas, goals, and concerns affect the formulation of principles. They also affect how health data are understood and managed by laws and regulations. Laws that regulate DH systems need a huge amount of health data. The role of technology, architecture, and innovations is to enable the realization of the HCDH system.

## Foundational Ideas and Goals in the HCDH Model

Foundational ideas and goals for HCDH are derived from the results of the literature analysis and concerns discussed in the DH: Concerns to Human Rights, Dignity, Privacy, and Autonomy section are shown in Table 2, together with corresponding ideas and goals, ideas of economic markets, and neoliberalism.

**Table 2:** Ideas and goals of human-centric digital health and neoliberal digital health.

Ideas and goals of human-centric digital health	Ideas and goals of neoliberal digital health
Maximization of health and wellness for all. Efficiency and cost saving cannot override human rights, privacy, and autonomy.	Maximization of own economic gain. Maximal efficiency and minimized costs.
Digitalization of health data that is ethically and lawfully collected.	Digitalization of all personal information.
Digital health Services are public goods. Public and private production of health services.	Private ownership of digital health services. Private production of health services.
Health data are not commodities and cannot be monetized.	Commodification and monetization of health data.
Personal health data are owned by the data subject and are not public goods or commodities. A person is the owner of digital products, such as digital patient twin derived from health data.	Private ownership of health data and digital products derived from it.
Strengthening of the role of the state. Strong privacy, dignity, and autonomy regulations	Deregulation and minimization of the role of the state. Weakening of privacy regulations.
Public and private organizations have responsibility for the consequences of the use and sharing of health data and digital products derived from data.	No responsibility for negative consequences.
Patient is the stakeholder in digital health.	Patient is a health consumer.
A person has the moral responsibility to promote good health behaviors and is not solely responsible for their own health and sickness.	Making a person or patient responsible for their own health and sickness management.

### Rights, Principles, and Responsibilities in HCDH

Essentially, human rights apply online just as they do offline [100]. There are fundamental questions in DH to be solved such as which rights should be absolute, and how privacy and health data should be understood. The authors state that rights presented in chapters 12 and 18 of the UNDHR and human dignity should be recognized as absolute rights. Human rights should be extended to cover sensitive data about humans and mental autonomy [39], and the extension “Nobody has the right to own a person physically, socially, mentally, or informationally” should be added. Furthermore, freedom of thought should be an absolute right [96]. Dignity should be understood as a human right, and it should also include social dignity. Without autonomy, we cannot exercise our other human rights; therefore, the right to mental autonomy is also needed. The authors state that the abovementioned human rights e cannot be balanced against other rights or others’ needs. Without the proposed extensions, public and private organizations have the power to modify our choices through nudges and other manipulation technologies. The authors also propose that personal digital self is not a commodity, and the person owns his or her digital copies (eg, DPT). This indicates that any form of digital self cannot be created, modified, or sold without a person’s consent.

HCDH also requires new responsibilities, which should be similar for public and private organizations collecting, processing, storing, and sharing health data. The current and widely used nonbinding moral responsibility to process health data fairly is insufficient. Responsibilities in DH should include the responsibility for negative consequences and harm caused to the data subject and society. Harm should be understood not only as economic loss, but instead it should cover health-related psychological and social harms and harms to autonomy [87]. Harms are difficult to predict and measure; therefore, they should be prevented proactively.

### Privacy and Health Data

According to researchers, currently it is almost impossible for a person to control the collection of health data and its flow across digital boundaries [90]. Therefore, it is necessary to extend the privacy concept to include behavioral privacy, privacy of thoughts and emotions, as well as metadata privacy. Furthermore, the authors state that privacy should be understood as the real-life ability to set personal rules (policies) regulating how health data are used in a situation, and as the legal duty for data collectors and processors to use data according to those rules. Furthermore, private information should be treated as private, and not as a resource that can be exploited by data companies [101]. According to researchers, privacy solutions using a fiduciary duty model and legal binding data rules and responsibilities should be used in the digital world [23,87,102], and the authors propose using them in HCDH. In DH, privacy should cover data collection, processing, and sharing of behavioral data, metadata, biomarkers, and person- or patient-generated data. It is also necessary to scrutinize why health data are used and by whom, and analyze the direct and indirect consequences of the possible misuse of health data (eg, discrimination, behavioral manipulation, and emotional manipulation). Available standards, such as the IEEE 7012 Standard for Machine Readable Privacy Terms, should be implemented.

In HCDH, the authors recommend the use of the wide approach for health data in such a way that it includes health-related behaviors, metadata, emotional data, inferred data, derived data, and self-generated data. Health data should also include induced products and simulations, such as a person’s digital self, digital twin, and DPT.

For HCDH, the authors propose the use of a special kind of ownership model where health data are personal properties and restricted public goods, that is, the person is the data owner, but health care providers, public health organizations, and professionals developing health care services, and researchers have the right to use it. Other uses of health data, such as the development of new health devices, products, and services and making economic gain, require a person’s consent. Furthermore, because people own their DH twin, it cannot be freely used, sold, or monetized by the data processor or other actors. This kind of personal ownership of health data corrects the existing data asymmetry (ie, those who have the ability to use health data also receive the biggest benefits) and power asymmetry by reducing the power data collectors can use over the data subject, and the lack of control [27]. It also supports the data subject’s autonomy.

In the future, personal wellness and health devices will generate a huge number of digital biomarkers and phenotypes used, stored, and shared by private industry. Digital biomarkers and phenotypes can also be linked with other personal data, resulting in potentially health-relevant data [103]. Currently, biomarkers are not classified as health data [15,55]. Furthermore, the digitization of past health-related data cannot be controlled by the data subject. To avoid risks for privacy and autonomy, the authors state that both digital biomarkers and past health data should be classified as health data.

### Laws and Regulations

According to available studies, current regulations are insufficient and unable to guarantee human rights, privacy, and autonomy in DH, especially in its multistakeholder digital ecosystems and future private-public coalitions, and against the network’s third-party data brokers and analytics companies [101]. Therefore, new laws and regulations are needed to make HCDH successful.

The authors propose that AI-based health algorithms should be defined as a medical device, and a law is needed to make certification of algorithms and AI applications using manipulative technology mandatory [45]. Furthermore, in DH, auditing, certification, and algorithmic verification should be made mandatory. It is also necessary to ban collection and surveillance through metadata and hidden reidentification [30]. New laws are required to regulate the use of self-tracking technologies and postmortem use of digital self and DPT [47]. Laws should extend transparency obligations for governments and industry, public authorities, and big data organizations collecting and processing health data.

The EU AI Act should be extended to prohibit AI systems that purposefully and materially manipulate a person’s preferences and generate harm. Furthermore, it is necessary to limit the use of facial recognition and ban algorithmic psychological, behavioral, emotional, and mind manipulation. According to the United Nations, a law is inevitable to protect people from unlawful or unnecessary surveillance, and to place human rights at the center of the regulation of digital technologies [104]. People also need the right to delete personal health data from commercial AI training databases [105]. Concerning AI algorithms, the principle of transparency is insufficient. For health AI applications, understandability of results, where they come from, and answers to questions about why and how data are used are needed.

### Technology and Architecture

In the digital world, there are many technological and algorithmic solutions and tools for maintaining privacy and anonymity, such as homomorphic encryption, differential privacy, blockchain technology, edge computing, secure multiparty computation, federated learning, tor lie services, dining cryptography, zero knowledge proof, noise adding, duty encryption, data poisoning, mobile edge computing, confidential computing, chatbots, privacy preserving, collaborative mining, ethical nudging, electronic consent, IP hiding, personal assistants for privacy, privacy policy analysis, risk estimation with AI, falsification of disclosed data at use device level, and collaborative machine learning [85,106], and more are being developed. Despite their benefits, the authors state that none of them alone can fulfill the needs for human rights, dignity, privacy, and autonomy in DH. Instead, a combination of different organizational, architectural, and technical solutions is needed. One possible solution is a public-private partnership ecosystem based on the fiducial duty model and the use of homomorphic encryption, federated learning, edge computing, or confidential computing [60].

## Design and Management of HCDH Ecosystems

**Figure 3:**
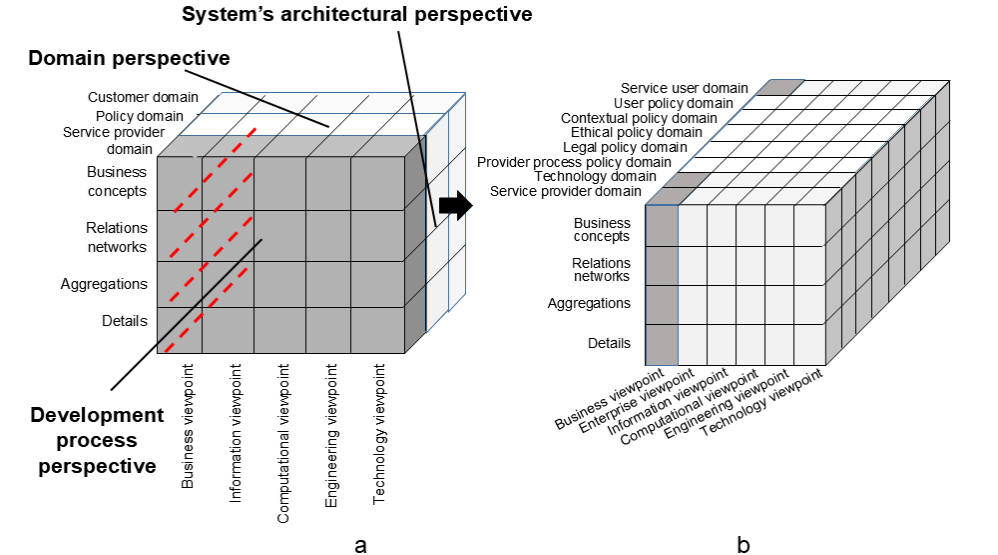
The International Organization for Standardization 23903 model and framework with focus on the policy domain (a) and its subdomains (b).

For designing and managing HCDH ecosystems, we must understand and formally as well as consistently represent multidisciplinary and dynamic systems in various contexts for enabling mapping between the different technologies, disciplines, methodologies, perspectives, intentions, and languages, among others. The solution is a system-oriented, architecture-centric, ontology-based, and policy-driven approach to transformed health ecosystems, using the universal type theory for abstraction and universal logic for representing relations. ISO 23903:2021 “Health informatics—interoperability and integration reference architecture—model and framework” provides a generic specification for managing interoperability and integration challenges in dynamic, complex, context-aware, and multidisciplinary health systems as “system of systems.” The system is represented using the generic component model [107,108]. A model is thereby defined as a representation of objects of a domain, their properties, relations, and interactions, enabling rational and active business in the represented domain (Figure 3). It consists of 3 dimensions: the domains involved in the business process, the granularity (composition and decomposition) of the system components, and the view of the evolutionary or development process. Thereby, the latter follows the ISO 10746 Reference Model Open Distributed Processing, defining the views of enterprise, information, computation, engineering, and technology; however, it is inevitably extended by the real-world business view.

The domain actors define the goals and objectives of the ecosystem. The behavior of the system is controlled by the policy domain, which must be refined into the following policy subdomains:

- the process policy domain, necessary for running the business process, separated into the service provider process policy domain (necessities for running the process) and the service user process policy domain (wishes and expectations) perspective
- the contextual policy domain, representing situational and intentional impacts
- the ethical policy domain, defining and enforcing humanistic, ethical, and moral requirements and solutions
- the legal policy domain, representing laws and regulations

The policies are formally represented using the policy ontology defined by the authors in ISO 22600 Privilege Management and Access Control.

Figure 3 formally represents all aspects shown in Figure 1, such as technology as domain information; communication technology, digital technologies, policies, information, data, implementation, and maintenance as viewpoints; as well as ideology, motivation, and beliefs as contexts.

## Challenges and Barriers in the HCDH Model

On the one hand, the expected positive impacts of DH seem to be so remarkable that the ongoing transformation is justifiable. On the other hand, DH should not overshadow human rights and cause the loss of dignity, privacy, and autonomy. In this paper, we have analyzed ideas and principles driving the evolution toward DH, and the impacts of this transformation on human rights, dignity, privacy, and autonomy through 6 views. On the basis of an extended literature analysis, we found that 1 possible transformation to DH uses ideologies of digital market economy and neoliberalism. Our analysis show that this kind of development, linked to the belief that technology has only 1 predefined direction, raises the risk of degradation of human rights, loss of dignity, privacy, and autonomy, and increasing inequality in health. In this paper, an alternative solution called “HCDH” is proposed. It respects human rights and dignity and maintains privacy and autonomy. In this model, a person or patient is a stakeholder, and not just a source of raw material or consumer. For HCDH, we have developed a system model; incorporated human-centric ideas, principles, and requirements; and proposed supporting laws. Cornerstones in HCDH are new rights that offer an individual a high level of autonomy and privacy, and a special kind of health data ownership, where health data are personal properties and restricted public goods. Our solution is also proactively prepared for privacy and autonomy concerns arising from new technological innovations, such as DPT and the use of AI for behavioral manipulation.

The HCDH model also has challenges. A major challenge is that guiding rules and principles of HCDH limit the ability of the data industry to collect, use, privatize, and monetize health data and use it for inferred or derived digital products and applications. As the current industry’s business model is based on free collection and use of data, they will voluntarily not stop the extensive collection of health data, push back its “ownership” to data patients and persons, and will not quit their money-making machine, thereby reducing their economic growth and ability to make innovations. Another problem is the wide definition used for health data. In the future, the findings of medical research and the better understanding of determinants for health can lead to an undesirable situation where most personal data would be health-relevant and also part of health data [55,61,109]. The industry may also argue that the expected benefits of the collection and use of health data legitimately pose possible negative consequences to human rights, dignity, autonomy, and privacy, and those consequences can be solved later [78]. The authors see this differently. In most real-life cases, innovations and development of new health products and services can be realized using AI-generated artificial data, and the current extensive, and wide collection of health data that violates human rights, privacy, and autonomy is unnecessary. Furthermore, the idea that negative consequences can be solved in the future is only a belief.

A possible barrier is that HCDH will be discarded by policy makers and data industry. In this case, the only protection persisting for the data subject or patient is to self-defend privacy, dignity, and autonomy at a local or personal level. There are already solutions for this approach, such as the creation of virtual unlinked personalities; hiding sources of data; developing apps, which enable the monitoring and filtering of data flow from personal devices; using personal intelligent agents to make data vanish; and using applications that generate poisoned information for data collectors and for AI training bases. However, the authors argue that wide range use of self-defending tools will have major negative impacts on the usability, quality, and reliability of health data repositories and it can distort results of AI applications [110]. Therefore, this kind of development is undesirable and should be avoided. Instead, governments and industry should offer people solutions that proactively prevent the misuse of health data by promoting human rights, dignity, privacy, and autonomy [70], such as the proposed HCDH.

DH should benefit people and patients in a way that is ethical, safe, secure, reliable, equitable, and sustainable. We must understand that if human rights, privacy, dignity, autonomy, and personal ownership of health data are lost, we will never get them back. Similar to Wong [39], we state that it is essential to understand that human rights, dignity, privacy, and autonomy should be core elements in DH. Without this, we are in danger of becoming holograms and digital slaves.

## Abbreviations

AI: artificial intelligence
BCI: brain computer interface
DH: digital health
DPT: digital patient twin
EU-GDPR: European Union General Data Protection Regulation
HCDH: human-centric digital health
UNDHR: United Nations Declaration of Human Rights
